# A Question of Custom

**Published:** 1899-04-22

**Authors:** 


### A QUESTION OF CUSTOM.

"Cantab" writes: Is it the custom for the senior physician at a small hospital to see all cases, whether surgical or not? Can he claim in purely surgical cases to pronounce whether an operation is advisable or not?

